# Acute Sleep Loss Upregulates the Synaptic Scaffolding Protein, Homer1a, in Non-canonical Sleep/Wake Brain Regions, Claustrum, Piriform and Cingulate Cortices

**DOI:** 10.3389/fnins.2020.00188

**Published:** 2020-03-13

**Authors:** Jingxu Zhu, Jennifer Hafycz, Brendan T. Keenan, Xiaofeng Guo, Allan Pack, Nirinjini Naidoo

**Affiliations:** Division of Sleep Medicine, Perelman School of Medicine, University of Pennsylvania, Philadelphia, PA, United States

**Keywords:** claustrum, *homer1a*, sleep deprivation, excitability, *in situ* hybridization

## Abstract

Homer proteins are a component of the post-synaptic density of neurons that are necessary for the maintenance and consolidation of behavioral state. The dominant negative protein homer1a is rapidly increased by neuronal activity and sleep loss. Homer1a knockout mice with globally absent homer1a have reduced ability to sustain wakefulness during the active period. It is not known whether homer1a is required globally or in very specific brain regions or neurons for its role in maintaining wake. In this study, we examined the expression of *homer1a*, an immediate early gene involved in intracellular signaling cascades, in mice subjected to extended wakefulness. We found that mice displayed increased expression of *homer1a* in the claustrum, a brain region thought to be involved in consciousness, as well as the cingulate and piriform cortices compared to non-sleep deprived mice. *In situ* hybridization (ISH) studies also indicate that *homer1a* is not induced in the known wake promoting regions with sleep deprivation, but is instead upregulated primarily in the claustrum and piriform cortex. Examination of *homer1a* expression levels with recovery sleep after sleep deprivation indicate that baseline *homer1a* expression levels were restored. Further, we have identified that homer1a is upregulated in excitatory neurons of the claustrum suggesting that homer1a promotes wakefulness through activating excitatory neurons. This work identifies regions previously unknown to be involved in sleep regulation that respond to acute sleep deprivation or enhanced waking.

## Introduction

Homer proteins function at the post-synaptic density as scaffolds, where they link several molecules important for cellular signaling. Specifically, homer1 functions as an adaptor to metabotropic glutamate receptors (mGluRs) as well as Shank proteins, PSD-95 expressed on NMDA receptors, and IP_3_ receptors expressed on the endoplasmic reticulum (Paschen and Mengesdorf, 2003; Piers et al., 2012). Homer1, therefore, has a role in synaptic plasticity and intracellular calcium signaling. Homer1 has three isoforms in mammals, homer1a, homer1b, and homer1c. Homer1a is classified as an immediate early gene and is the short form of homer1, lacking the C-terminal coiled coil domain, while homer1b and homer1c are long forms. Homer1a competes with the long forms of homer1 in a dominant negative manner, disrupting the signaling connections between homer1b, homer1c, and their binding partners.

Homer1a is a known molecular correlate of sleep loss (Nelson et al., 2004; Maret et al., 2007; Mackiewicz et al., 2008; Naidoo et al., 2012). Homer1a mRNA is upregulated under sleep-deprived conditions in mouse cortices and hypothalamic tissue (Nelson et al., 2004; Mackiewicz et al., 2007; Maret et al., 2007). We have previously reported that global knockout of homer1a in mice leads to an inability to maintain long bouts of wakefulness, suggesting a role for homer1a in maintenance of wakefulness (Naidoo et al., 2012). Further, it has been shown that homer1a is required for the alteration of synapses during sleep (Hu et al., 2010). More recent data indicate that homer1a protein moves into the synapse during sleep and is responsible for synaptic downscaling (Diering et al., 2017). This evidence suggests that homer1a function is crucial for proper sleep-wake behaviors and synaptic homeostasis. Despite this, little is known about the direct role of homer1a on sleep and wake behavior or its mechanism of action.

In order to better understand the role of homer1a as an immediate early gene product in sleep and wake, we used *in situ* hybridization to assay *homer1a* mRNA expression across the mouse brain under conditions of acute sleep loss compared to that in sleeping mice. Previous studies have examined *homer1a* expression following six or more hours of sleep deprivation. We report in this study that *homer1a* is not robustly induced in the known wake promoting regions following three or less hours of sleep loss, but is instead upregulated primarily in the claustrum, piriform and cingulate cortices.

In addition, following recovery sleep *homer1a* expression levels in these neuronal groups are restored to baseline levels. Finally, we have found that sleep-loss induced *homer1a* expression in claustral neurons co-localizes with that of CAMKIIα, a marker for excitatory neurons. Together, these results identify the claustrum as a novel brain region that demonstrates changes in *homer1a* expression in response to very short periods of sleep deprivation (SD) and could therefore be involved in the regulation of sleep and wake.

## Materials and Methods

### Mice

All experiments were performed on male mice. C57/BL6 mice were 8 weeks of age ± 1 week. All mice were maintained on 12hr light/dark cycle in a sound attenuated recording room, temperature 22–24°C. Food and water were available *ad libitum*. Animals were acclimated to these conditions for 7 days before beginning any studies. All animal experiments were performed in accordance with the guidelines published in the NIH Guide for the Care and Use of Laboratory Animals and were approved by the University of Pennsylvania Animal Care and Use Committee.

### Sleep Deprivation

Mice were sleep deprived (SD) for either 1 h or 3 hours. Deprivation was initiated at 12 pm (ZT5) for 1 h or 10 am (ZT 3) for 3 h. Deprivations were performed through gentle handling following a 5 day acclimatization period for handling procedures as previously described (Naidoo et al., 2008). Undisturbed spontaneously sleeping mice were used as diurnal controls at 1 pm (SC).

### Recovery Sleep

Since 3-hour sleep deprivation produced the greatest change in Homer1a expression only mice that underwent 3-hour sleep deprivation, (10 am-1 pm) were allowed 3-hour recovery sleep (1 pm-4 pm). Mice were sacrificed at the end of the recovery sleep (RS) period. Diurnal undisturbed sleeping controls were obtained at 4 pm.

### Tissue Collection

The solutions used for *in situ* hybridization (ISH) were prepared using 0.1% diethylpyrocarbonate (DEPC) water. Mice were perfused transcardially with 0.9% saline, followed by 4% paraformaldehyde immediately following undisturbed sleep, sleep deprivation or the recovery sleep period. Brains were collected and post-fixed in 4% paraformaldehyde solution with RNase inhibitor at 4°C. After 24 h brains were moved to 30% sucrose with RNase inhibitor at 4°C. Brains were sectioned coronally in a cryotome at 40 micrometers, collected in 1:6 series with free-floating solution (1xPBS with 0.05% and RNase inhibitor), and stored at 4°C until processed for ISH, RNAscope or IHC. A complete set of brain sections representing the entire brain were used.

### Digoxigenin (DIG) Labeled Probes *in situ* Hybridizations Procedure

Sense and antisense DIG-labeled RNA probes were respectively synthesized from full length cDNA clones of Homer1a (or Galanin for studies of VLPO (cDNA clone MGC:54666 IMAGE:6476459 plasmid, Invitrogen) using T7 (anti-sense) and SP6 (sense) polymerases (Roche). The probes were stocked at −80°C until used for hybridization. All procedures were performed at room temperature unless a different temperature was stated. Free-floating coronal sections were fixed with 4% PFD for 30 min and then permeabilized with 0.4% Triton-X100 overnight. The sections were incubated with 0.1M triethanolamine and 0.25% acetic anhydride for 10 min, and then rinsed with 2X saline–sodium citrate (SSC) solution. Sense and antisense probes were denatured at 85°C for 3 min and then on ice for 5 min before mixed with hybridization buffer (1:1000) with 0.25mg/ml tRNA (Roche) to make hybridization solutions. The sections were hybridized with hybridization solutions for 16-18 hours overnight at 55°C in a humidified chamber. The sections were washed with 4×, 2×, and 1× SSC solution each for 30 min with three time changing and 0.1X SSC solution at 60°C for 1 h. To detect the signals, the sections were incubated with anti-digoxigenin-AP antibody (1:3000, Roche 11093274910) for 48 h at 4°C and then developed with NBT/BCIP (1:100, Sigma) for 16–18 h at 4°C. The sections were mounted using an aqueous mounting solution - Vecte Mount AQ (Vector, H5501). Some sections were counterstained with GNG2 (Abcam, ab19825) antibody (see below IHC) before mounting to examine the colocalization of Homer1a and GNG2 at claustrum. Digital brightfield images were obtained on a Leica microscope (Leica).

### Quantitative Analysis of Dioxigenin (DIG) ISH Images

To compare the ISH changes of homer1a after sleep deprivation and recovery sleep, three groups of animals (SD, SC, and SR) that were treated equally for perfusion, cutting and DIG ISH were used. Sections from a 1 in 12 series through the claustrum between Bregma 1.4 to 0.2 mm were used for DIG ISH. Three sections from the claustrum and piriform cortex were analyzed per animal. All images were captured using the 20× objective on a Leica DM5500 microscope at the same setting. Calculation of the percent area occupied by Homer1a ISH positive area fraction was performed using NIH ImageJ (National Institutes of Health, Bethesda, MD, United States) software domain (Papadopulos et al., 2007). To analyze *Homer1a* expression on the images, NIH ImageJ (National Institutes of Health, Bethesda, MD, United States) software domain was used (Papadopulos et al., 2007). Briefly, every individual color image was converted to 8-bit format image with the Image/Type/8-bit command. The 8-bit gray scale image was adjusted with the Image/Adjust/Threshold Command for threshold 120. From Analyze/Measure command, the percentage of total area that the positive *homer1a* ISH occupies was automatically displayed on the result sheet. These data were used for statistical analyses.

### RNAscope *in situ* Hybridization (ISH)

RNAscope *in situ* hybridization was performed in 40 μm floating brain tissue sections using the method modified from Grabinski et al. (2015) to detect and visualize the co-localization of Homer1a with CamKIIα, GAD1 or Gng2. The RNAscope Multiplex Fluorescent Reagent Kit (323100) and RNAscope probes [mouse Homer1a (432441-C2), Gng2 (462481), GAD1 (400951-C3), CamKIIα (445231)] were from Advanced Cell Diagnostics (ACD, Hayward, CA, United States). For the modified manual RNAscope experiments free-floating coronal sections were fixed with 4% PFD for 30 min. After washing with PBS, the sections were treated with 3% H_2_O_2_ for 30 min and then permeabilized with 0.4% Triton-X100 overnight. Sections were mounted on superfrost plus slides (Fisher Scientific) and dried at 60°C for 30 min. After cooling to room temperature, the slides were pretreated with III reagent for 20 min at 40°C. Hybridization with double probes (Gng-C1 and Homer1a-C2) or triple probes (CanKIIα-C1, Homer1a-C2, and GAD1-C3) were performed for 2 hours at 40°C, followed by amplification. TSA fluorophores labeling was carried out using TSA Fluorescein for C1, TSA Cy3 for C2 and TSA Cy5 for C3 respectively. Fluorescent images were captured using a Leica confocal microscope (SP-5 AOBS, Leica).

### Immunohistochemistry (IHC) and Immunofluorescence (IF) Staining Procedures

To identify different known wake-active neurons, IHC was performed using a commercially available kit according to manufacturer’s instructions (ABC Immunodetection kit, Vector Laboratories, Burligame, CA, United States) for Orexin-A (1:1000, sc-8070) in the lateral hypothalamus, Tyrosine Hydroxylase (TH, 1:1000, AB152, Millipore) in locus coeruleus. Histidine Decarboxylase (HDC, 1:2000, 2263B260-1, EuroProxima) in tuberomammillary nucleus (TMN). Briefly, 40 μm brain sections were treated with 0.3% H_2_0_2_ in PBS for 30 minutes and then blocked with 0.4% normal donkey serum (NDS) and 1% BSA for 2 h. The primary antibody was applied overnight at room temperature washed and then incubated with biotinylated secondary antibody for 60 min. The reaction was developed with an avidin-biotin complex reaction and the signals were detected by DAB. Digital brightfield images were recorded on a Leica microscope (Leica).

Double immunofluorescence staining (Naidoo et al., 2011) was used to characterize the waking c-Fos response in claustrum, orexinergic neurons and LC. Latexin (1:100, PA5-28534, Invitrogen), Orexin-A and TH were labeled with Alexa fluor 594 (Invitrogen) by secondary antibodies and c-Fos (1:1000, ab208942, Abcam) was labeled with Alexa fluor 488 (Invitrogen) by secondary antibody. Fluorescent images were captured using a Leica confocal microscope (SP-5 AOBS, Leica).

### Punches and Q-PCR

For these experiments, mice were euthanized by cervical dislocation and brains were quickly removed, rapidly frozen on dry ice, and stored at −80°C. Micropunches of the brain were performed in a cryostat as described (Palkovits, 1983; Palkovits et al., 1985). Five brain regions, the claustrum, piriform cortex, lateral hypothalamus, tuberomammillary nucleus, and locus coeruleus, were dissected according to the mouse brain atlas from Allen Institute for Brain Science (Bakker et al., 2015; Lein et al., 2007). Claustrum (CLA) was dissected using 1.2 mm i.d. Harris Uni-Core Micro-Punch (Whatman) from four 300 μm sections between approximately 1.6 to 0.4 mm Bregma. Piriform cortex (PIR) was dissected from four 300 μm sections between approximately 0.4 to −0.8 mm Bregma using 1.2 mm i.d. micropunch. Lateral hypothalamus (LHA) was dissected from four 300 μm sections between approximately −0.8 to −2.0 mm Bregma using 1.2 mm i.d. micropunch. Tuberomammillary nucleus (TMN) was dissected from two 300 μm sections between approximately −2.0 to −2.6 mm Bregma using 1.2 mm i.d. micropunch. Locus coeruleus (LC) was dissected from two 200 μm sections between approximately −5.2 to −5.6 mm Bregma using 1.0 mm i.d. micropunch.

RNA extraction was performed using RNAqueous-Micro Total RNA Isolation Kit (Invitrogen) and on-column DNase digestion was performed with RNase-Free DNase Set (Qiagen). RNA concentration and purity were measured by Nanodrop 1000 spectrophotometer. Thirty-six samples were randomly chosen from the five regions for evaluation of RNA integrity (RIN) using Agilent 2100 Bioanalyzer RNA 6000 Nano chip. All samples measured had a RIN value above 8.0.

cDNA was synthesized from RNA using High-Capacity RNA-to-cDNA kit (Applied Biosystems), and quantitative real-time PCR assays were performed using TaqMan assays for the following gene targets: Fos (Mm00487425_m1), Arc (Mm00479619_g1), BDNF (Mm01334043_m1), Homer1 (Mm00516275_m1), Homer1a (customized AIT95T0, derived from nucleotides 1342–2140); region specific markers: Gng2 (Mm00726459_s1) (marker for CLA), Hcrt (Mm01964030_s1) (marker for LHA), Hdt (Mm00456104_m1) (marker for TMN), Th (Mm00447557_m1) (marker for LC); house-keeping genes Hprt (Mm01545399_m1), and Tbp (Mm00446971_m1). RT-PCR reactions were performed using an Applied Systems 7900HT Fast Real-Time PCR System with 10ng of cDNA in each reaction. For the gene assays that may detect genomic DNA, namely Arc, Homer1a, Gng2 and Hcrt, no-RT control experiment (reverse transcriptase was removed from the cDNA synthesis reaction to detect any residual amplification from genome DNA template) was run for each sample to confirm the effectiveness of DNase-treatment. Relative gene expression was calculated using the deltaCt method (Vandesompele et al., 2002; Schmittgen and Livak, 2008), where the Ct value of each target gene was corrected using the geometric mean of the Ct values of the two housekeeping genes. Analyses were performed on negative deltaCt (−ΔCt) values, such that more positive values indicate higher expression.

## Statistical Methods

### q-PCR Analyses and Statistics

To evaluate differences across conditions, we performed a joint hypothesis test simultaneously assessing whether there were any differences in PCR values across three *a priori* pairwise comparisons of interest: (i) sleep deprivation vs. 3-hour spontaneous sleep control, (ii) sleep recovery vs. 6-hour control, and (iii) sleep deprivation vs. sleep recovery. This test is similar to an ANOVA, providing an overall comparison of >2 groups, but allows for separate control groups. Given multiple genes in each region, statistical significance of the joint hypothesis test was based on a Hochberg corrected threshold (Hochberg, 1988). If the joint null hypothesis was rejected, we subsequently evaluated each pairwise comparison to determine which differences were driving the overall effects, again with a Hochberg correction for multiple comparisons (Hochberg, 1988; Huang and Hsu, 1994). Results are presented as model-estimated means and 95% confidence intervals within each group.

### *In situ* Hybridization (ISH) Analysis

Prior to analysis, average values were obtained within each section and region, resulting in 3 separate measurements in the claustrum or piriform cortex for each mouse. Given this, comparisons of percentage area among conditions were performed using a mixed model analysis of variance (ANOVA) with a random intercept term to account for multiple measures per animal. This analysis assessed the global null hypothesis of no differences among conditions (control, sleep deprivation, recovery sleep). If this global null hypothesis was rejected (*p* < 0.05), we performed subsequent pairwise comparisons between conditions; statistical significance in pairwise comparisons was based on a Hochberg corrected threshold (Hochberg, 1988). Percentage area values were natural log transformed for analyses in order to satisfy parametric assumptions; for interpretability, results are presented as group-specific geometric means and 95% confidence intervals.

## Results

### Acute Sleep Loss Induces Homer1a Expression in the Claustrum, Cingulate and Piriform Cortices

We have previously reported that *homer1a* mRNA expression is increased in the brain with 6 h of sleep loss (Mackiewicz et al., 2007) and that homer1a is required for the maintenance of wakefulness (Naidoo et al., 2012). To examine the spatial distribution of changes in expression of *homer1a* mRNA in the brain following sleep loss, mice at two months of age were subjected to either one or three hours of sleep deprivation beginning at ZT 5 (noon) or ZT 3 (10 am), respectively. Thus, sleep deprivation ended at the same diurnal time in both groups. With this acute sleep deprivation paradigm, we were able to assess more immediate molecular changes that occur with sleep loss, particularly in the form of homer1a as an immediate early gene product. Mice were perfused immediately following sleep deprivation and brain slices were subjected to *in situ* hybridization for the Homer1a gene. Diurnal sleeping controls were perfused at the same time points.

**FIGURE 1:**
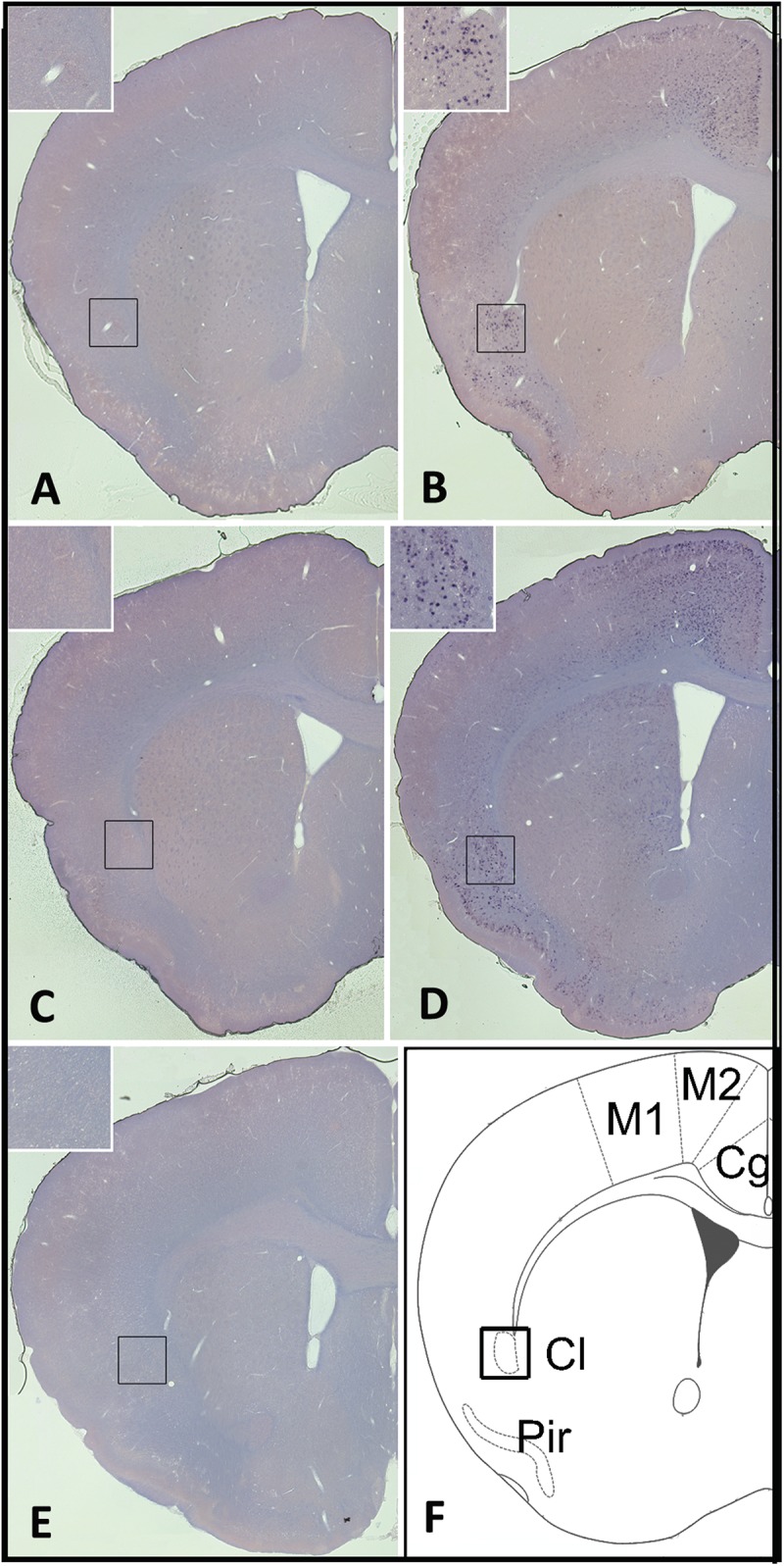
Sleep loss induces *homer1a* expression in the claustrum, cingulate and piriform cortices. **(A)** *Homer1a* expression levels in 1hr sleep control claustrum enlarged; **(B)** *Homer1a* expression levels following 1 h sleep deprivation (SD), claustrum enlarged; **(C)** *Homer1a* expression levels in 3 h sleep diurnal control, claustrum enlarged; **(D)** *Homer1a* expression levels following 3 h SD; **(E)** *In situ* hybridization sense control; **(F)** diagram of brain slice showing pertinent regions. Bar size 1 mm.

**FIGURE 2:**
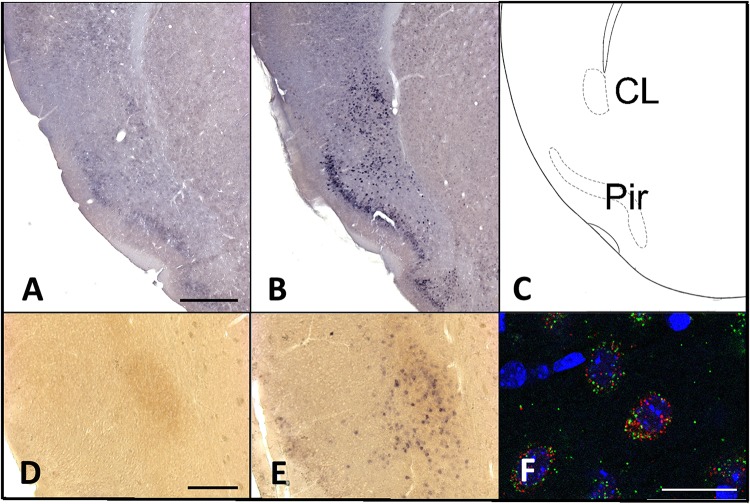
Increases in *homer1a* mRNA are localized to the claustrum and piriform cortex. **(A)** Homer1a *in situ* 3 h sleep control (SC); **(B)** *Homer1a in situ* hybridization 3 h sleep deprivation (SD); **(C)** corresponding zoomed-in atlas image; **(D)** *Homer1a in situ* and GNG2 immunostain in 3 h sleep control; **(E)** *Homer1a in situ* and GNG2 immunostain in 3 h SD; **(F)** *Homer1a* (red), and *Gng2* (green) RNAscope with DAPI (blue). Bar size A: 500 μm, D: 250 μm, F: 25 μm.

Overall, *homer1a* mRNA expression is increased following sleep deprivation in the claustrum, cingulate, and piriform cortices as revealed by *in situ* hybridization (Figure 1). The changes in expression levels of *homer1a* mRNA were more robust in these same regions and in the motor cortex following three-hours of sleep deprivation; the highest expression was localized to the claustrum and the piriform cortex (Figure 1). Further, immunostaining of Gng2, a protein enriched in the claustrum was used as a claustral marker (Wang et al., 2017), coupled with *in situ* hybridization to *homer1a* in matched brain sections, confirmed localization of *homer1a* expression to the claustrum (Figure 2E). As the Gng2 antibody is not particularly robust, since clearly immunostained puncta are not observed, we also confirmed *homer1a* localization to the claustrum using RNAscope double *in situ* hybridization with mRNA for Gng2 (Figure 2). Here we found that the *homer1a* and *Gng2* mRNA probes co-localize to the same neurons (Figure 2F).

### Homer1a Expression Is Localized to Excitatory Neurons in the Claustrum

**FIGURE 3:**
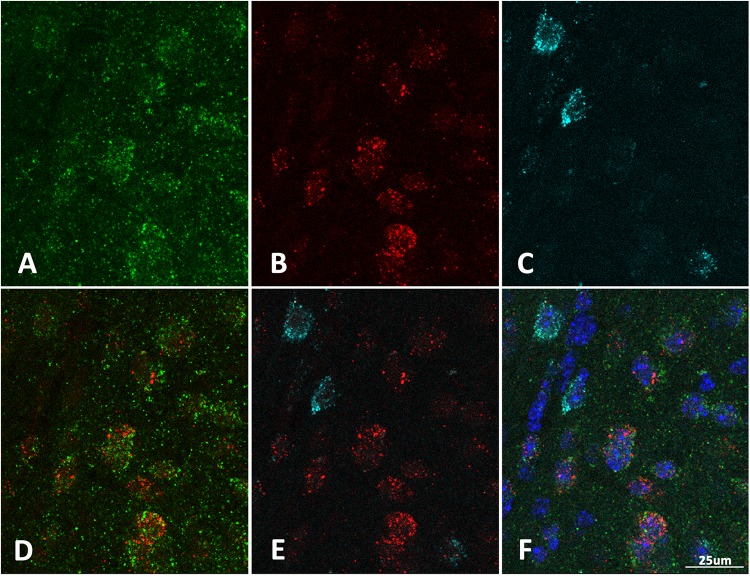
Homer1a expression is localized to excitatory neurons in the claustrum. **(A)** Immunostain for CAMKIIα (green); **(B)** Immunostain for Homer1a (red); **(C)** Immunostain for GAD67 (cyan); **(D)** CAMKIIα and Homer1a overlap; **(E)** GAD67 and Homer1a merge; **(F)** Merge CAMKIIIα, GAD67, Homer1a, and DAPI (blue). Bar size: 25 μm.

While the majority of claustrum neurons are excitatory, the region consists of a heterogeneous population of both excitatory and inhibitory neurons. To determine if there was differential expression of homer1a in a subpopulation of claustrum neurons, we carried out *in situ* hybridization for homer1a and co-immunostaining with either CAMKIIα or GAD67 as markers for excitatory and inhibitory neurons, respectively. Our immunostaining revealed that the increased homer1a protein expression in sleep-deprived mice co-localizes with excitatory CAMKIIα-positive cells (Figure 3). This suggests that the expression of homer1a with extended wakefulness could influence cellular excitability.

### Homer1a Expression Is Not Induced in Canonical Sleep/Wake Brain Regions Following Sleep Deprivation

**FIGURE 4:**
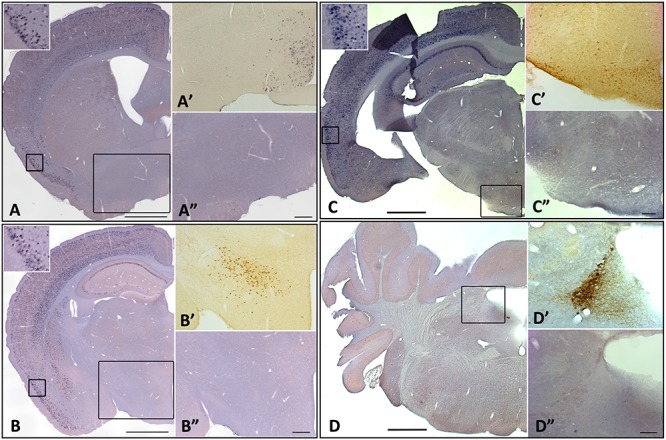
*Homer1a* expression is not induced in canonical sleep/wake brain regions following sleep deprivation. *Homer1a* ISH shown as positive control in small inset for each section. Brain sections illustrating. **(A)** Ventrolateral preoptic nucleus, VLPO, (large inset); **(A’)** Galanin ISH as a marker for the VLPO; **(A”)** *Homer1a in situ* in VLPO; **(B)** lateral hypothalamus, LHA, (large box); **(B’)** Orexin immunostain as a marker for the LHA; **(B”)** *Homer1a in situ* in enlarged LHA; **(C)** Tuberomamillary nucleus, TMN, (large box); **(C’)** Histamine immunostain as a marker for the TMN; **(C”)** *Homer1a in situ* in enlarged TMN; **(D)** Locus coeruleus, LC, (large box); **(D’)** Tyrosine Hydroxylase immunostain as marker for the LC; **(D”)** *Homer1a in situ* in enlarged LC. Bar size **A,B,C,D**: 1 mm, **A”,B”**: 250 μm, **C”,D”**: 100 μm.

**FIGURE 5:**
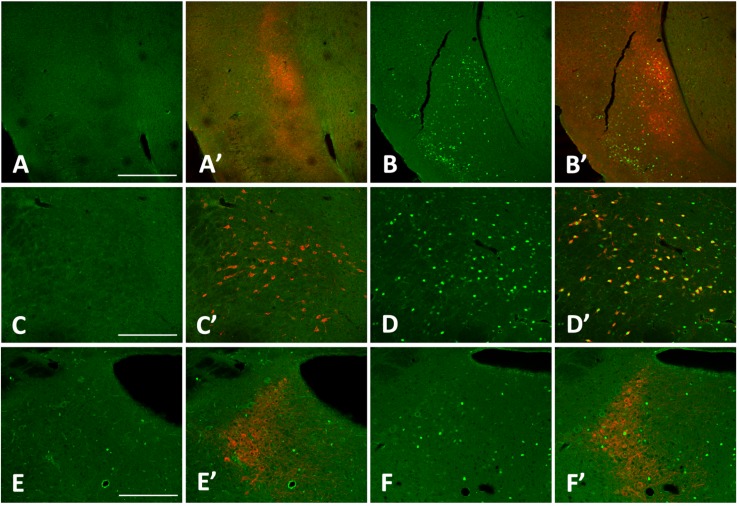
cFos levels increase in the claustrum, lateral hypothalamus (LHA), and locus coeruleus (LC) with 3 h sleep deprivation (SD) compared to sleeping control (SC) animals. **(A)** 3 h SC cFos staining; **(A’)** 3 h sleep control with cFos and Latexin as marker for the claustrum; **(B)** 3 h SD cFos staining; **(B’)** 3 h SD with cFos and Latexin; **(C)** 3 h SC cFos staining; **(C’)** 3 h sleep control with cFos and Orexin as marker for the LHA; **(D)** 3 h SD cFos staining; **(D’)** 3 h SD with cFos and Orexin; **(E)** 3 h SC cFos staining; **(E’)** 3 h sleep control with cFos and tyrosine hydroxylase (TH) as marker for the LC; F) 3 h SD cFos staining; **(F’)** 3 h SD with cFos and TH. Bar size **A:** 500 μm; **C,E**: 250 μm.

Interestingly, there were no apparent changes in *homer1a* expression by *in situ* hybridization in classically studied wake-promoting and sleep-promoting brain regions, including the noradrenergic neurons of the locus coeruleus (LC), orexin neurons of the lateral hypothalamus (LHA), the histaminergic neurons in the tuberomammillary nucleus (TMN), or the sleep promoting ventrolateral pre-optic area (VLPO) (Figure 4). To verify that the traditional wake active neuronal populations were in fact activated during enforced wakefulness/sleep deprivation we carried out c-Fos immunostaining and found that c-Fos was expressed with 3 h of sleep deprivation in the locus coeruleus, orexin neurons in the LHA and histaminergic neurons in the TMN, but there was no increase in homer 1a expression (see Figure 5).

### Homer1a Expression Is Reduced With Recovery Sleep

**FIGURE 6:**
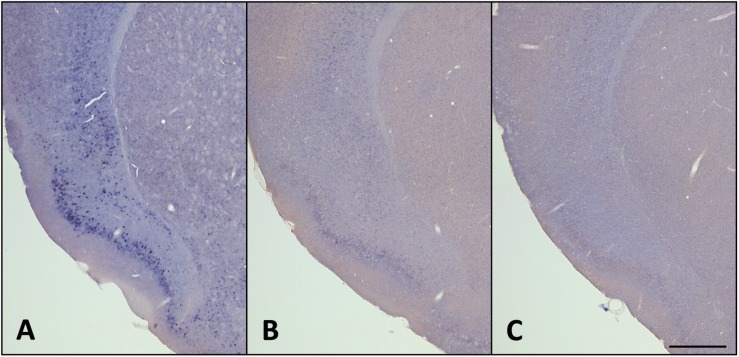
*Homer1a* expression is reduced with recovery sleep (RS). **(A)** 3 h SD; (B) 3 h SD + 3 h RS; **(C)** SC. Bar size: 500 μm. See also Supplementary Table S1.

In order to determine if *homer1a* mRNA expression is down-regulated with sleep, we subjected mice to 3 h of sleep deprivation followed by a 3-hour period of recovery sleep. These sleep-deprived mice were perfused immediately following 3 h of recovery sleep. There was a separate group of normally sleeping mice that were sacrificed at the same diurnal time. *Homer1a* expression levels in mice that had been allowed recovery sleep were similar to *homer1a* expression levels in the undisturbed diurnal spontaneously sleeping control mice in the brain regions of interest (motor cortices, piriform cortices, claustrum) as determined by *in situ* hybridization (see Figure 6).

Quantification of the *homer1a in situ* hybridization signal in the claustrum and piriform cortex indicate that *homer1a* mRNA is significantly altered with behavioral state in these regions (see Supplementary Table S1). Specifically, *homer1a* in the claustrum of young mice is increased with sleep deprivation compared to undisturbed sleep (1.91 [0.69, 5.23] vs. 0.27 [0.10, 0.75]% area; *p* = 0.007) and decreased with recovery sleep compared to sleep deprivation (0.34 [0.12, 0.92] vs. 1.91 [0.69, 5.23]% area; *p* = 0.017). There was no difference in *homer1a* during undisturbed sleep and recovery sleep in the claustrum (*p* = 0.780). In the piriform of these young mice, we again observed increased *homer1a* during sleep deprivation compared to undisturbed sleep (5.79 [2.48, 13.53] vs. 0.44 [0.19, 1.03]% area; *p* < 0.0001). While there is trending evidence of a reduction in values during recovery sleep compared to sleep deprivation (1.85 [0.79, 4.33] vs. 5.79 [2.48, 13.53]% area; *p* = 0.063), *homer1a* expression was still elevated in the piriform following recovery sleep compared to undisturbed sleep (1.85 [0.79, 4.33] vs. 0.44 [0.19, 1.03]% area; *p* = 0.019).

### Homer1a Expression in Mice Following Sleep Loss Is Recapitulated by q-PCR

Changes in *homer1a* expression with sleep loss were also confirmed by quantitative PCR using punches of the key regions identified by *in situ* hybridization.

**FIGURE 7:**
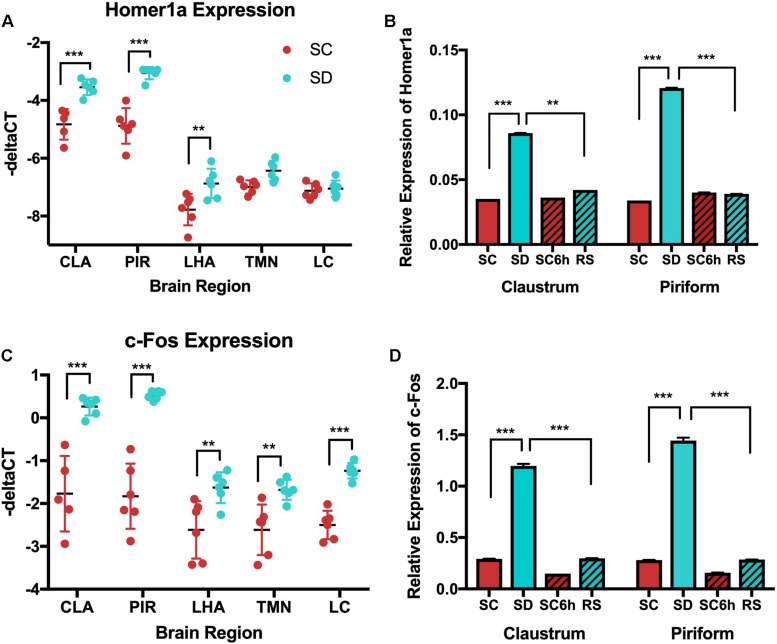
QPCR shows increased *homer1a* expression in the claustrum (CLA) and piriform cortex (PIR) with sleep deprivation (SD) and *c-fos* increases in response to SD across all regions. **(A)** *Homer1a* qPCR across all brain regions in SC and SD conditions; **(B)** *Homer1a* qPCR across all behavioral conditions in claustrum and piriform. *Homer1a* expression is increased with SD in claustrum (*p* < 0.001) and piriform cortex (*p* < 0.0001) SC (sleep control); SD (sleep deprived); RS (recovery sleep); LHA (lateral hypothalamus);PIR (piriform cortex); CLA (claustrum); TMN (Tuberomammillary nucleus); LC (Locus coeruleus). *C-fos* is increased in response to SD in all regions and is reduced with recovery sleep in the claustrum and piriform. C) qPCR of *c-Fos* shows increased *c-Fos* in response to SD in all regions tested (*p* < 0.001); D) *c-Fos* qPCR across sleep deprivation (SD), 3 h sleep control (SC), 6 h diurnal sleep control (SC6h), and recovery sleep (RS) in young mice, (*p* < 0.001). See also Supplementary Figure S1 and Supplementary Tables S2–S4.

As a first step, we directly examined differences in *homer1a* expression levels via qPCR across the different regions during undisturbed sleep (Figure 7A and Supplementary Table S2). We observed significant differences across regions (*p* < 0.0001); *homer1a* expression in the claustrum and piriform cortex was significantly greater than expression in other canonical wake regions (LH, TMN, LC).

When examining the effects of sleep loss, as for *in situ* hybridization in Figure 1, *homer1a* transcript assessed by qPCR is significantly increased with 3 h of sleep deprivation compared to undisturbed sleep in the claustrum (−3.54 [−3.97, −3.12] vs. −4.83 [−5.29, −4.36] –ΔCT; *p* = 0.0004) and piriform cortex (−3.05 [−3.36, −2.75] vs. −4.88 [−5.19, −4.58] –ΔCT; *p* < 0.0001) (see Figure 7B and Supplementary Table S3]. To ensure that we had successfully punched out the claustrum, we assayed for transcript levels of *Gng2*, a marker for the claustrum and determined that *Gng2* did not change with behavioral state (Supplementary Figure S1). *Homer1a* was not increased in the LC or TMN by qPCR, recapitulating the *in situ* hybridization observation (*p* = 0.759 and *p* = 0.137, respectively). On the other hand, qPCR reveals a small, but significant increase in *homer1a* expression in the LHA with sleep deprivation compared to 3-hour sleep controls (−6.87 [−7.23, −6.51] vs. −7.78 [−8.14, −7.42] –ΔCT; *p* = 0.0013; Figure 7 and Supplementary Tables S2, S3). When examining statistical evidence for differential *homer1a* responses to sleep deprivation across these regions (Supplementary Table S3), there was a significant interaction (*p* < 0.001; Supplementary Table S3). Increases in *homer1a* with sleep deprivation were generally larger in the claustrum and piriform cortex than canonical wake regions.

We further confirmed our *in situ* hybridization findings of reduced *homer1a* expression in the claustrum following recovery sleep. In both the claustrum and piriform cortex, *homer1a* values were significantly reduced after recovery sleep when compared to values during sleep deprivation, and values return to levels seen in control animals sleeping undisturbed for 6 h (see Supplementary Table S3 and Figure 7B). Supporting the differential responses to sleep loss and recovery, statistical interaction tests show regional differences in the comparisons between recovery sleep and sleep deprivation (*p* ≤ 0.002) (Supplementary Table S3).

### QPCR Shows Increases in *c-fos* Following Sleep Deprivation in All Wake-Active Neurons

Similarly, we utilized qPCR to evaluate *c-fos* expression in wake active brain regions (LC, TMN, lateral hypothalamus) as well as in the claustrum and piriform cortex following sleep deprivation and sleep recovery. In contrast to results for *homer1a*, *c-fos* was expressed at similar levels across interrogated brain regions (*p* = 0.082; Figures 7C,D, see Supplementary Table S4).

When examining differences across behavioral conditions, we observed significant increases in *c-fos* with sleep deprivation in all brain regions (see Supplementary Table S4 and Figures 7C,D). While all regions showed increased *c-fos* with sleep loss compared to undisturbed sleep, there were significant regional differences in these mice (*p* = 0.002) (Figure 7C and Supplementary Table S4); increases in *c-fos* with sleep deprivation based on qPCR were generally larger in the claustrum and piriform cortex. Similar data was observed by c-Fos immunostaining of these regions (Figure 5).

Further, *c-fos* expression levels were reduced with recovery sleep compared to values after sleep deprivation in all regions (Figure 7D, see Supplementary Table S4). Unlike results for *homer1a*, where changes with sleep deprivation returned to levels seen in controls during sleep recovery, there remained residual increases in *c-fos* after sleep recovery compared to the 6-hour sleep control mice in all regions except the LC (*p* = 0.120). While results with recovery sleep were qualitatively similar across regions, there was again evidence of significant regional effects on the magnitude of the difference between recovery sleep and sleep loss (*p* = 0.0001) (Supplementary Table S4). Results are again supported by immunostaining of these regions (see Figure 5).

### Plasticity Marker Arc but Not BDNF Is Upregulated in Claustrum and Piriform Cortex With Sleep Deprivation

**FIGURE 8:**
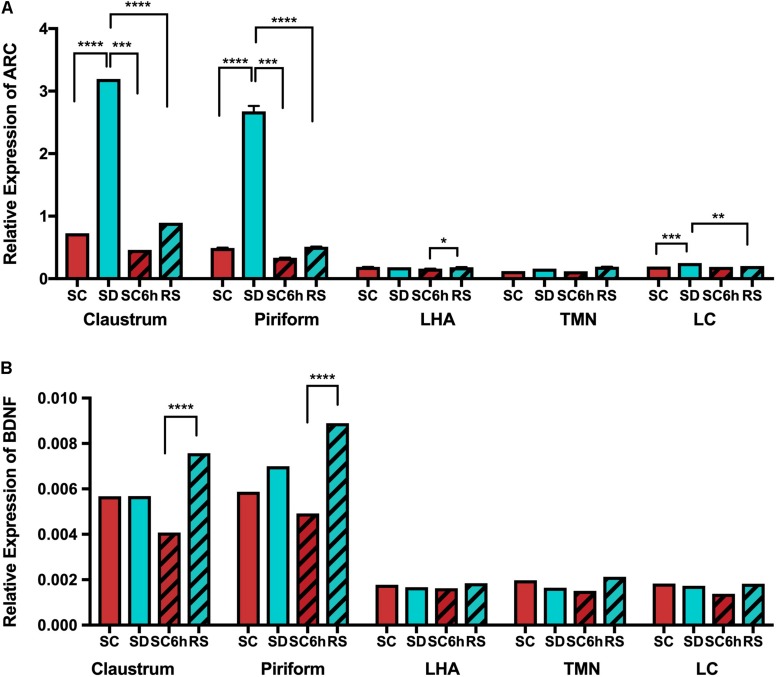
QPCR shows increased *Arc* expression in claustrum and piriform cortex with SD. **(A)** *Arc* expression is shown across all conditions and brain regions. **(B)** *BDNF* expression is also shown across all brain regions and conditions.

We also examined the expression of the plasticity genes *Arc* and *BDNF* by q-PCR in the claustrum, piriform cortex, LC, TMN and lateral hypothalamus following 3 h of sleep deprivation and 3 h of recovery sleep (see Supplementary Table S5). *Arc*, like *homer1a*, is increased in the claustrum and piriform cortex in mice subjected to 3 h of sleep deprivation when compared to undisturbed sleeping controls (all *p* < 0.0001; Figure 8). Similarly, *Arc* values were significantly reduced with recovery sleep compared to sleep deprivation in these regions (all *p* < 0.0001; Supplementary Table S5), although there remained residual increases (*p* < 0.009). *Arc* expression is not increased in the lateral hypothalamus or TMN with sleep deprivation, however, there were small, but significant increases in *Arc* transcript in the LC with sleep deprivation (*p* = 0.0046) that was reduced on average with recovery sleep (Supplementary Table S5 and Figure 8). We found no changes in *BDNF* transcript levels in any of the brain regions examined with sleep deprivation or recovery sleep when compared to undisturbed diurnal controls (Figure 8).

## Discussion

In this study, we examined the effect of acute sleep loss on *homer1a* expression levels and spatial distribution in the brains of mice. The goal was to identify the early molecular changes that occur with sleep loss, particularly with *homer1a* as an immediate early gene product, as opposed to the changes that occur with extended sleep loss, as described by Maret et al. (2007). Intriguingly, we found that *homer1a* expression increased in the claustrum, piriform, and cingulate cortices of these mice and to a much larger extent than in other brain regions. Importantly, *homer1a* expression was low and changed little in classically studied wake-active neuronal groups following sleep deprivation. Our *in situ* hybridization results were confirmed with q-PCR. Namely, *homer1a* expression increased in the claustrum, piriform cortex, and cingulate cortex following sleep deprivation. This increase was not present in classically studied wake-active regions, i.e., the tuberomammillary nucleus or the locus coeruleus. Further, following recovery sleep after sleep loss, *homer1a* expression levels were reduced in the claustrum and piriform cortex. QPCR data also demonstrated that *homer1a* expression levels were reduced to the same extent with recovery sleep in both the claustrum and piriform cortex.

*Homer1* encodes constitutively expressed long-form proteins that serve as scaffolds in the post synapse, connecting important signaling molecules such as Shank proteins, mGluRs, and IP_3_ calcium receptors on the endoplasmic reticulum (Paschen and Mengesdorf, 2003; Piers et al., 2012). The short-form homer1a, which lacks the C-terminal coiled-coiled domain, is a dominant negative that prevents dimerization and hence breaks the scaffold connections (Kammermeier and Worley, 2007). Its transcription is increased with neuronal activity (Bottai et al., 2002) and has been thought of as an immediate early gene. Our data indicate, however, that there is a dissociation between increases in c-Fos, which also occurs with neuronal activity (Hoffman et al., 1993) and increases in *homer1a* expression. Some areas such as claustrum show increase in both whereas other neuronal groups (TMN, LC, and orexin neurons) show only increases in c-fos but not *homer1a*.

Homer1a is involved in important synaptic processes, specifically synaptic remodeling during sleep. It has been proposed that at sleep onset homer1a protein is targeted to synapses where it binds mGluR1/5 and remodels or activates mGluR1/5 signaling to drive synaptic down-scaling (Diering et al., 2017). Previously, our work has shown that mice lacking homer1a globally are unable to maintain wakefulness. Mice lacking homer1a had short bouts of wakefulness and not the long bouts that occur in the early part of the lights off period (Naidoo et al., 2012). The neuronal basis of this effect on behavior is not known. The results presented here demonstrate that *homer1a* increases with extended wakefulness in the claustrum, as well and the piriform and cingulate cortices, which have not been previously implicated as key regulators of sleep and wake. The cingulate cortex, however, is known to play a role in emotional processing, learning and memory. Recent studies have revealed that abnormal connectivity in the anterior cingulate cortex is associated with insomnia (Yan et al., 2018). Further, the piriform is mostly involved in olfaction. It is known that during slow-wave sleep, the piriform cortex becomes hypo-responsive to odor stimulation and functional connectivity to cortical regions is enhanced (Barnes and Wilson, 2014). In mice, it is unsurprising that both these regions demonstrate synaptic activity in the form of *homer1a* expression, though the role of *homer1a* expression in these regions is not exactly known and whether there is a role for homer in connectivity within these regions is unknown.

What we found particularly interesting were the changes in *homer1a* expression levels in the claustrum, as very little is known about the function of this region. The claustrum is an enigmatic brain region whose function is unknown. The claustrum is a thin, irregular, laminar neuronal structure located beneath the inner surface of the neocortex, near the insula, with extensive reciprocal projections to numerous cortical and subcortical regions, such as the prefrontal cortex, primary sensory cortices, thalamus, and reticular formation (Smith and Alloway, 2010; Torgerson and Van Horn, 2014; Torgerson et al., 2015; Wang et al., 2017). Based on its structure and connectivity, Crick and Koch proposed that the claustrum integrates and binds together different cortical inputs, such as color, smell, sound, and touch, into a single unifying experience in consciousness (Crick and Koch, 2005). The claustrum is highly integrated with most cortical regions. Several qualitative studies indicate that the claustrum has topographical and reciprocal connections with the motor, premotor, orbitofrontal, prefrontal, parietal, cingulate, temporal, visual, perirhinal, and entorhinal cortices (Edelstein and Denaro, 2004; Druga et al., 2014; Zingg et al., 2014). As a result of these connections, it has been postulated to regulate conscious experiences (Crick and Koch, 2003, 2005; Blumenfeld, 2014). More recent quantitative studies using AAV tracers have confirmed that the claustrum has strong reciprocal and bilateral connections with prefrontal and cingulate areas as well as strong reciprocal connections with the ipsilateral temporal and retrohippocampal areas (Wang et al., 2017). The latter study suggests that the claustrum may play a crucial role in a variety of cognitive processes. Recent data indicate that several claustrum neurons wrap around the entire mouse brain (Koch, 2017), further emphasizing its connectivity to the whole brain. The claustrum has been hypothesized to be a saliency detector (Remedios et al., 2014; Smythies et al., 2014), identifying novel stimuli as well as a synchrony detector, integrating oscillations from other brain regions (Smythies et al., 2012). Case studies have shown that electrical stimulation of the claustrum results in a loss of consciousness (Koubeissi et al., 2014) and that claustrum damage is associated with the duration of loss of consciousness (Chau et al., 2015).

Importantly, the claustrum consists of a heterogeneous population of excitatory and inhibitory neurons (Kitanishi and Matsuo, 2017). Our data indicate that the increase in *homer1a* expression with extended wakefulness is specific to excitatory CAMKIIα-positive neurons. This suggests a potential mechanism to explain the role of *homer1a* in the maintenance of wakefulness. Because the claustrum is thought to be involved in synchronizing oscillations from the cortex (Smythies et al., 2014), it is possible that *homer1a* expression in excitatory cells potentiates the development of gamma oscillations in the claustrum. Gamma oscillations are low amplitude, high frequency brain waves associated with wake and rapid eye movement (REM) sleep. Consistent with this idea, a recent study by Renouard et al. suggests that the claustrum may activate the cortex during REM sleep (Renouard et al., 2015). It is conceivable that increased *homer1a* expression in the claustrum strengthens gamma oscillations thereby producing a mechanism that sustains wakefulness. Further research manipulating claustrum neurons and *homer1a* levels in these neurons is needed to understand whether gamma oscillations are altered and could regulate sleep and wake behavioral states. More recent data also suggest a role for the claustrum in insomnia, as genes found to be associated with insomnia using GWAS were also found to contain genes that are expressed in the claustrum (Jansen et al., 2019).

In conclusion, *homer1a* is specifically upregulated in the claustrum and piriform cortex, but not in classic wake-active neuronal groups. Recovery sleep following sleep deprivation reduces *homer1a* expression in these brain regions back to control levels. Because *homer1a* has many functions (Szumlinski et al., 2006; Leber et al., 2017) and binds to different intracellular signaling molecules, it is crucial to understand the role of *homer1a* in regulating sleep-wake behaviors. Our results raise the possibility that the role of *homer1a* in sustaining wakefulness is mediated by its effect on excitatory neurons in the claustrum.

## Data Availability Statement

The raw data supporting the conclusions of this manuscript will be made available by the authors, without undue reservation, to any qualified researcher.

## Ethics Statement

The methods and study protocols were approved in full by the Institutional Animal Care and Use Committee of the University of Pennsylvania and conformed to the revised National Institutes of Health Office of Laboratory Animal Welfare Policy.

## Author Contributions

NN designed the experiments, analyzed data, and wrote the manuscript. AP edited the manuscript. JZ and XG performed the experiments. JH performed the experiments, analyzed the data, and wrote the manuscript. BK conducted all the statistical analyses for the project.

## Conflict of Interest

The authors declare that the research was conducted in the absence of any commercial or financial relationships that could be construed as a potential conflict of interest.
