# Supplementary material for: Acute Sleep Loss Upregulates the Synaptic Scaffolding Protein, Homer1a, in Non-canonical Sleep/Wake Brain Regions, Claustrum, Piriform and Cingulate Cortices
Source: Front Neurosci. 2020 Mar 13;14:188. doi: 10.3389/fnins.2020.00188 (PMC7083128; doi:10.3389/fnins.2020.00188)
Supplement: Supplementary file 1 [file Data_Sheet_1.PDF]

SI Table Titles:

Table S1: ISH quantification of Homer1a in behavioral conditions within mice. Related to Figure 6
Table S2: Regional Differences in qPCR data at baseline. Related to Figure 7.

Table S3: qPCR results for Homer1a across behavioral conditions in mice. Related to Figure 7.

Table S4: PCR results for c-Fos across behavioral conditions in mice. Related to Figure 7

Table S5: PCR results for ARC and BDNF across behavior conditions. Related to Figure S2

Supplemental Information and Titles:

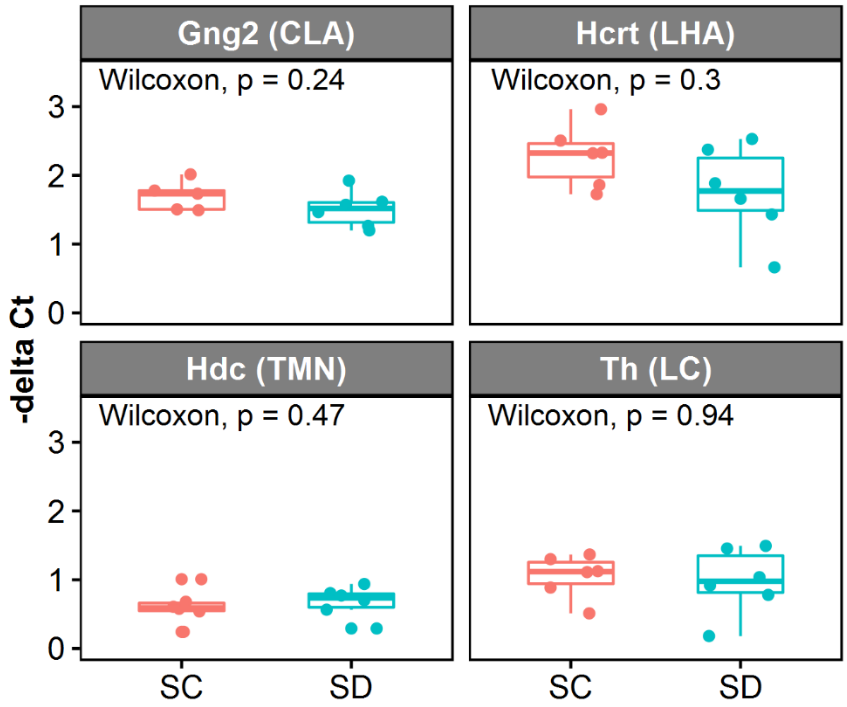

Supplemental Figure 1: Specific markers for each region did not change with state, shown are Gng2 for the claustrum, hypocretin/orexin for the lateral hypothalamus, histidine decarboxylase for the TMN, and tyrosine hydroxylase for the LC. Related to Figure 7.

Table S1: ISH quantification of Homer1a in behavioral conditions within mice. Related to Figure 6

Region	Age	Geometric Mean (95% CI) % Area†			ANOVA p-value	Pairwise p-values		
		Sleep Control	Sleep Deprivation	Sleep Recovery		SD vs. SC	SR vs. SC	SR vs. SD
Claustrum	2-3 Month	0.27 (0.10, 0.75)	1.91 (0.69, 5.23)	0.34 (0.12, 0.92)	**0.0138**	**0.0078**	0.7803	**0.0172**
Piriform	2-3 Month	0.44 (0.19, 1.03)	5.79 (2.48, 13.53)	1.85 (0.79, 4.33)	**0.0001**	**<0.0001**	**0.0186**	0.0631

†Analyses performed on log transformed % area, results presented as model estimated geometric mean (equal to the exponentiated average of log-transformed values) for interpretability; Significant interaction (p<0.05), ANOVA (p<0.05) or pairwise comparisons (after Hochberg correction) presented in **bold**.

Table S2: Regional Differences in qPCR data at baseline. Related to Figure 7.

Gene	Mean (95% Confidence Interval) –ΔCt Value					ANOVA p-value‡
	Claustrum	Piriform Cortex	Lateral Hypothalamus	Tubero-mammillary Nucleus	Locus Coeruleus	
Homer1a	-4.84 (-5.25, -4.40)	-4.88 (-5.27, -4.49)	-7.78 (-8.17, -7.39)	-6.99 (-7.38, -6.60)	-7.12 (-7.51, -6.73)	**<0.0001**
c-Fos	-1.77 (-2.38, -1.16)	-1.83 (-2.39, -1.27)	-2.61 (-3.17, -2.06)	-2.61 (-3.17, -2.06)	-2.50 (-3.06, -1.94)	0.0820
ARC	-0.46 (-0.85, -0.07)	-1.03 (-1.39, -0.67)	-2.41 (-2.77, -2.06)	-3.05 (-3.41, -2.69)	-2.37 (-2.72, -2.01)	**<0.0001**
BDNF	-7.46 (-7.79, -7.13)	-7.41 (-7.71, -7.11)	-9.13 (-9.44, -8.83)	-8.98 (-9.29, -8.68)	-9.09 (-9.40, -8.79)	**<0.0001**

Table S3: qPCR results for Homer1a across behavioral conditions in mice. Related to Figure 7.

Region	Mean (95% Confidence Interval) Homer1a –ΔCt Value				Joint p-value‡	Pairwise Comparisons		
	3-hour Sleep Control	Sleep Deprivation	6-hour Sleep Control	Recovery Sleep		SD vs. SC3hr	RS vs. SC6hr	SD vs. RS
CLA	-4.83 (-5.29, -4.36)	-3.54 (-3.97, -3.12)	-4.79 (-5.21, -4.37)	-4.57 (-4.99, -4.15)	**0.0008**	**0.0004**	0.4443	**0.0020**
PIR	-4.88 (-5.19, -4.58)	-3.05 (-3.36, -2.75)	-4.64 (-4.95, -4.34)	-4.68 (-4.98, -4.37)	**<0.0001**	**<0.0001**	0.8719	**<0.0001**
LHA	-7.78 (-8.14, -7.42)	-6.87 (-7.23, -6.51)	-6.94 (-7.30, -6.58)	-6.93 (-7.29, -6.57)	**0.0034**	**0.0013**	0.9855	0.8011
TMN	-6.99 (-7.52, -6.46)	-6.43 (-6.96, -5.90)	-7.07 (-7.60, -6.54)	-6.59 (-7.12, -6.06)	0.2511	0.1374	0.1929	0.6711
LC	-7.12 (-7.47, -6.78)	-7.05 (-7.40, -6.70)	-8.01 (-8.35, -7.66)	-7.18 (-7.52, -6.83)	**0.0018**	0.7592	**0.0021**	0.5971

‡p-value from joint hypothesis test evaluating any significant pairwise differences between SD vs. SC3hr, SR vs. SC6hr, or SD vs. SR; Significant p-values in joint comparison (p<0.05) or pairwise tests (after Hochberg correction) shown in **bold**.
Abbreviations: CLA: Claustrum; PIR: Piriform; LHA: lateral hypothalamus; TMN: tuberomammillary nucleus; LC: locus coeruleus.

Table S4: PCR results for c-Fos across behavioral conditions in mice. Related to Figure 7.

Region	Mean (95% Confidence Interval) c-Fos –ΔCt Value				Joint p-value‡	Pairwise Comparisons		
	3-hour Sleep Control	Sleep Deprivation	6-hour Sleep Control	Sleep Recovery		SD vs. SC3hr	RS vs. SC6hr	SD vs. RS
CLA	-1.77 (-2.29, -1.25)	0.26 (-0.21, 0.74)	-2.74 (-3.22, -2.26)	-1.75 (-2.22, -1.27)	**<0.0001**	**<0.0001**	**0.0061**	**<0.0001**
PIR	-1.83 (-2.28, -1.38)	0.53 (0.07, 0.98)	-2.66 (-3.11, -2.20)	-1.81 (-2.26, -1.36)	**<0.0001**	**<0.0001**	**0.0119**	**<0.0001**
LHA	-2.61 (-3.03, -2.20)	-1.63 (-2.05, -1.21)	-3.38 (-3.79, -2.96)	-2.72 (-3.13, -2.30)	**0.0001**	**0.0023**	**0.0306**	**0.0010**
TMN	-2.61 (-3.09, -2.13)	-1.68 (-2.10, -1.20)	-3.36 (-3.84, -2.88)	-2.56 (-3.04, -2.08)	**0.0006**	**0.0095**	**0.0221**	**0.0140**
LC	-2.50 (-2.81, -2.19)	-1.24 (-1.55, -0.92)	-2.79 (-3.10, -2.48)	-2.45 (-2.76, -2.14)	**<0.0001**	**<0.0001**	0.1200	**<0.0001**

‡p-value from joint hypothesis test evaluating any significant pairwise differences between SD vs. SC3hr, SR vs. SC6hr, or SD vs. SR; Significant p-values in joint comparison (p<0.05) or pairwise tests (after Hochberg correction) shown in **bold**. *Abbreviations:* CLA: Claustrum; PIR: Piriform; LHA: lateral hypothalamus; TMN: tuberomammillary nucleus; LC: locus coeruleus.

Table S5: PCR results for ARC and BDNF across behavior conditions. Related to Figure S2.

Gene	Region	Mean (95% Confidence Interval) –ΔCt Value				Joint p-value‡	Pairwise Comparisons		
		3-hour Sleep Control	Sleep Deprivation	6-hour Sleep Control	Sleep Recovery		SD vs. SC3hr	SR vs. SC6hr	SD vs. SR
ARC	CLA	-0.46 (-0.90, -0.02)	1.68 (1.28, 2.08)	-1.12 (-1.52, -0.72)	-0.16 (-0.56, 0.24)	**<0.0001**	**<0.0001**	**0.0022**	**<0.0001**
	PIR	-1.03 (-1.40, -0.65)	1.42 (1.04, 1.79)	-1.59 (-1.96, -1.21)	-0.85 (-1.22, -0.47)	**<0.0001**	**<0.0001**	**0.0088**	**<0.0001**
	LHA	-2.41 (-2.60, -2.22)	-2.44 (-2.63, -2.25)	-2.65 (-2.84, -2.45)	-2.44 (-2.63, -2.25)	0.2729	0.8165	0.1215	0.9572
	TMN	-3.05 (-3.43, -2.67)	-2.63 (-3.00, -2.25)	-3.08 (-3.45, -2.70)	-2.39 (-2.77, -2.01)	**0.0378**	0.1132	**0.0147**	0.3711
	LC	-2.37 (-2.54, -2.20)	-2.00 (-2.17, -1.83)	-2.40 (-2.57, -2.23)	-2.30 (-2.47, -2.13)	**0.0094**	**0.0046**	0.3972	**0.0171**
BDNF	CLA	-7.46 (-7.84, -7.08)	-7.46 (-7.81, -7.11)	-7.94 (-8.28, -7.59)	-7.04 (-7.39, -6.70)	**0.0112**	0.9948	**0.0012**	0.0921
	PIR	-7.41 (-7.66, -7.17)	-7.16 (-7.40, -6.91)	-7.67 (-7.91, -7.42)	-6.81 (-7.06, -6.57)	**0.0004**	0.1441	**<0.0001**	0.0503
	LHA	-9.13 (-9.34, -8.93)	-9.23 (-9.43, -9.02)	-9.26 (-9.47, -9.06)	-9.08 (-9.29, -8.88)	0.5558	0.5183	0.2071	0.3133
	TMN	-8.98 (-9.33, -8.64)	-9.24 (-9.59, -8.90)	-9.37 (-9.72, -9.03)	-8.87 (-9.22, -8.53)	0.1530	0.2784	0.0439	0.1277
	LC	-9.09 (-9.33, -8.86)	-9.17 (-9.41, -8.94)	-9.51 (-9.74, -9.27)	-9.10 (-9.33, -8.86)	0.0556	0.6165	0.0192	0.6383

‡p-value from joint hypothesis test evaluating any significant pairwise differences between SD vs. SC3hr, SR vs. SC6hr, or SD vs. SR; Significant p-values in joint comparison (p<0.05) or pairwise tests (after Hochberg correction) shown in **bold**. *Abbreviations:* CLA: Claustrum; PIR: Piriform; LHA: lateral hypothalamus; TMN: tuberomammillary nucleus; LC: locus coeruleus.
